# Prophylactic melatonin for delirium in critically ill patients: A systematic review and meta-analysis with trial sequential analysis

**DOI:** 10.1097/MD.0000000000031411

**Published:** 2022-10-28

**Authors:** Wenqing Yan, Chen Li, Xin Song, Wenqiang Zhou, Zhi Chen

**Affiliations:** a Medical Department of Nanchang University, Nanchang, Jiangxi, China; b Department of Emergency, Jiangxi Provincial People’s Hospital, Nanchang, Jiangxi, China; c Department of Traumatology, Jiangxi Provincial People’s Hospital, Nanchang, Jiangxi, China; d The First Affiliated Hospital of Nanchang Medical College, Nanchang, Jiangxi, China.

**Keywords:** critically Ill patient, delirium, melatonin, melatonin receptor agonist, meta-analysis, prevention

## Abstract

Up to 80% of patients in the intensive care unit (ICU) suffer from delirium. Studies on the preventative use of melatonin in the ICU have produced mixed results. We performed a systematic review and meta-analysis to evaluate whether early administration of melatonin reduces the prevalence of delirium in critically ill patients.

**Methods:**

We searched Medline, Embase, and the Cochrane Library for randomized controlled trials comparing melatonin or melatonin agonists to placebo in ICU setting. The population included adult patients in the ICU. The primary outcome was the prevalence of delirium. Secondary outcomes included duration of delirium, delirium-free day, serum melatonin concentration, need for sedation, duration of mechanical ventilation, hospital and ICU length of stay (LOS), all-cause mortality, sleep quality, and adverse events. Trial sequential analysis (TSA) was performed on the primary outcome to prevent the risk of random error and multiplicity phenomenon as a result of repeated significance testing across all the included trials.

**Results:**

Twelve trials with a total of 2538 patients were analyzed. When all trials were pooled, the incidence of delirium in ICU patients who received melatonin was significantly lower than in those who received placebo (risk ratio, 0.77; 95% confidence interval: 0.61–0.96; *I*^2^ = 56%). There were no significant differences in secondary outcomes including duration of delirium, duration of mechanical ventilation, ICU LOS, hospital LOS, and mortality. TSA indicated that Z-curve crossed the traditional boundary, but did not cross the monitoring boundary for benefit, which indicated that it is still inconclusive that melatonin affects the incidence of delirium.

**Conclusions:**

This meta-analysis found that early administration of melatonin may result in a decreased delirium prevalence in critically ill patients. However, the sensitivity analysis of high-quality studies did not support this finding. In addition, TSA demonstrated that the result may have false-positive error. Therefore, this finding should be interpreted with caution. Further studies are needed to examine the effectiveness of prophylactic melatonin on the prevalence and duration of ICU delirium in the future.

## 1. Introduction

Delirium is a condition characterized by acute organic brain dysfunction with fluctuating disturbances in attention and cognition.^[1]^ Up to 80% of patients in the intensive care unit (ICU) develop delirium.^[2–4]^ Delirium is associated with unfavorable outcomes, including higher mortality, longer time on the ventilator, and longer time in the ICU and hospital.^[2,5]^ Currently, there are no effective pharmacological agents for delirium prevention in ICU.^[2,6]^

Melatonin, an endogenous pineal gland hormone, exhibits anti-inflammatory immunomodulatory properties and regulates circadian rhythm.^[7]^ Multiple studies have demonstrated impaired melatonin secretion in critically ill patients, which may be associated with the development of sleep deprivation and delirium.^[8]^ Accordingly, there has been growing interest in the effects of exogenous melatonin in ICU settings. Studies evaluating melatonin as a prophylactic agent have suggested that melatonin could play an important role in preventing delirium in critically ill.^[2,7,8]^

However, evidence of the clinical effectiveness of prophylactic melatonin in delirium prevention is mixed. Previous systematic reviews and meta-analyses^[9,10]^ have shown that melatonin reduced the incidence of delirium in ICU patients, although they were limited by sample size. Since then, additional randomized controlled trials (RCTs) evaluating the effect of melatonin on delirium prevention in critically ill patients have been conducted.^[7,11]^ The latest RCT,^[7]^ a large sample size, demonstrated that enteral melatonin initiated within 48 hours of ICU admission did not reduce the prevalence of delirium compared with placebo. With new evidence available, an updated systematic review of the literature and meta-analyses is required. This systematic review and meta-analysis aimed to evaluate whether the early administration of melatonin reduces the prevalence of delirium.

## 2. Methods

The protocol was prospectively registered at the International Prospective Register of Systematic Reviews (registration number: CRD42022326352). Reporting of findings was guided by the Preferred Reporting Items for Systematic reviews and Meta-Analyses.^[12]^

### 2.1. Eligibility criteria

We included RCTs that met the following criteria: population: adult patients (≥ 18 years old) who were admitted to the ICU; intervention: melatonin or melatonin receptor agonists (ramelteon) compared with a placebo, standard of care, or no intervention (referred to as control); and the RCT reported at least 1 of the following outcomes: delirium prevalence, duration of delirium, and delirium-free days. Observational studies, ecological studies, case series, case reports, reviews, abstracts, editorials, comments, letters to the editor, and unpublished studies will not be included. Studies involving healthy volunteers were not eligible. Studies without full text were excluded.

### 2.2. Literature search

A systematic search of PubMed, EMBASE, and the Cochrane Library was performed from database inception through to April 15, 2022 by 2 authors (WQY and CL). In addition, we conducted a hand search of references obtained from identified studies and known meta-analyses. All languages are included as long as there is an English abstract or full-text article. The actual search strategy was available in Supplement 1, Supplemental Digital Content, http://links.lww.com/MD/H803.

### 2.3. Study selection

Two authors (WQY and XS) separately screened all retrieved citations by reviewing their titles and abstracts. Full-text articles were retrieved if either of the authors considered the abstract potentially suitable. Next, the same authors independently assessed each study’s eligibility based on the inclusion criteria. Disagreements were resolved by discussion or consensus with a third author (ZC).

### 2.4. Data extraction

Two reviewers (CL and WQZ) independently extracted individual study data using a predefined data extraction form. Any discrepancies in data extraction were discussed until a consensus was reached. If consensus could not be reached between the 2 independent reviewers, a third reviewer (ZC) made the final decision. Extracted data included the following: publication details, study design details, inclusion criteria of subjects, sample size, patient demographics, type and dosage of the used agent, delirium assessment method, and assessments of the outcomes. When a study reported follow-up at different periods, outcomes with the longest follow-up were extracted.

### 2.5. Assessment risk of bias

Two reviewers evaluated studies for risk of bias using a previously piloted standardized form and the Cochrane Risk of Bias 2 tool^[13]^ for RCTs. The following domains were assessed: bias arising from the randomization process, bias due to deviations from intended interventions, bias due to missing outcome data, bias in the measurement of the outcome, and bias in the selection of the reported result. The overall risk of bias for each included study was categorized into low risk of bias, some concern of bias, or high risk of bias.

### 2.6. Clinical outcomes and subgroup analyses

The primary outcome was delirium prevalence. Secondary outcomes were delirium-free days, delirium duration, serum melatonin concentration, need for sedation, duration of mechanical ventilation (MV), hospital and ICU length of stay (LOS), all-cause mortality, sleep quality, and adverse events. The longest follow-up period was used for mortality. Subgroup analyses of primary outcome were performed. If the information was available, we planned to conduct subgroup analyses based on older patients (age ≥ 60 years), different types of patients (surgical patients or medical patients), different types of diagnosis, and different types of melatonin (melatonin or ramelteon).

### 2.7. Data synthesis

For all continuous outcomes, the mean and standard deviation were sought. median and interquartile range were converted to mean and standard deviation using established methods.^[14,15]^ Data were represented as relative risk (RR) for dichotomous outcomes and mean difference (MD) for continuous outcomes with 95% confidence intervals (CI). A random-effects model was used, and statistical significance was set at *P* < .05 for all analyses. Heterogeneity degree across included studies was explored by inspecting the forest plots and calculating *I*^2^ statistic, with an *I*^2^ of > 50%, demonstrating a statistically significant heterogeneity. To ensure the robustness of our primary outcome, sensitivity analysis was performed based on studies with low risk of bias. The risk of publication bias was assessed by visual interpretation of funnel plots. Meta-analyses were conducted using the Review Manager 5.4 software (RevMan, The Cochrane Collaboration, 2020). Results for outcomes for which meta-analysis was deemed inappropriate because of an insufficient number of studies or clinical or statistical heterogeneity were reported in narrative form.

Trial sequential analysis (TSA) was performed on the primary outcome to prevent the risk of random error and multiplicity phenomenon as a result of repeated significance testing across all the included trials. TSA tests the credibility of the ascertained results by combining both an estimation of information size (a cumulative sample size of included trials) with an adjusted threshold of statistical significance for the cumulative meta-analysis. Meta-analysis monitoring boundaries (Trial Sequential Monitoring Boundaries) and the required information size were quantified, alongside diversity-adjusted information size and adjusted 95% confidence intervals. The required information size was calculated based on a 2-sided sequential analysis fixed-effect model with 5% risk of type 1 error, 80% of power, relative risk reduction of 20% and 31.66% of incidence of control arm.

## 3. Results

### 3.1. Study selection and study characteristics

**Figure 1.**
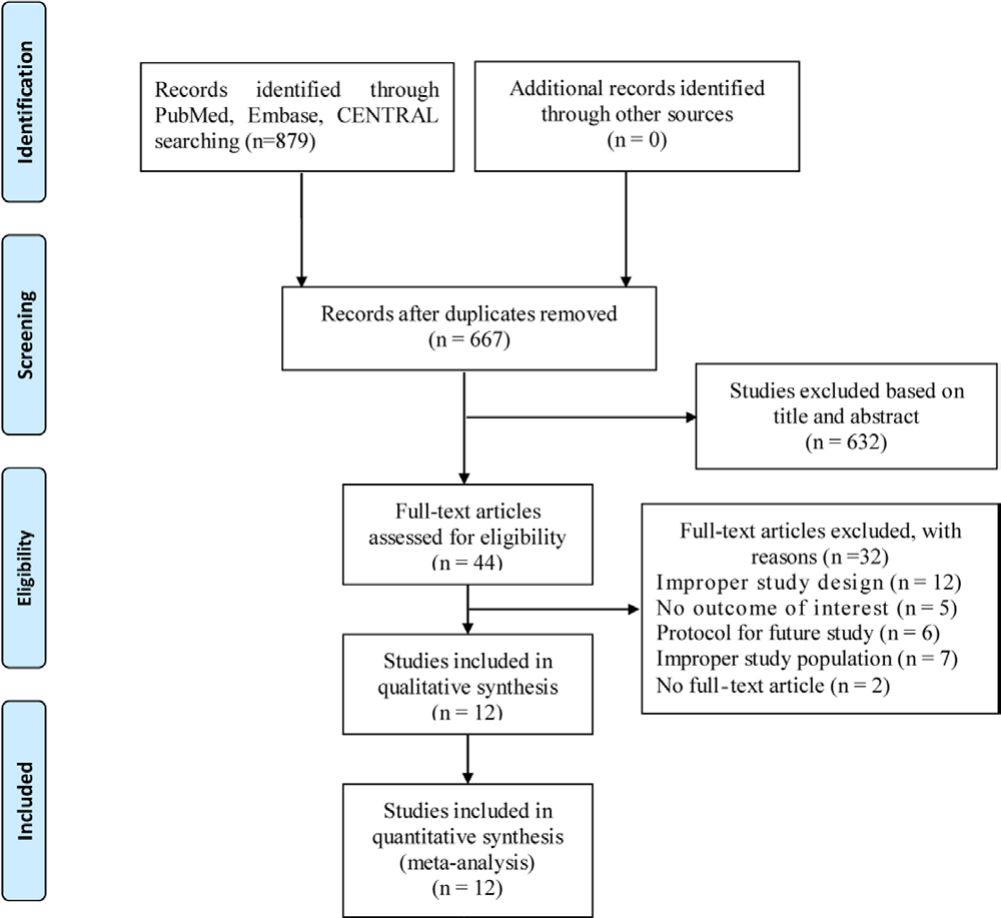
Study flow diagram.

Twelve trials,^[7,11,16–25]^ including 2538 patients, were evaluated and eligible for the meta-analysis. The flow diagram for study inclusion is shown in Figure 1. Characteristics of the studies included in the meta-analysis was shown in Table 1. All trials provided relevant data on 1 or more targeted outcomes suitable for the final analysis. The included trials were published between March 2011^[24]^ and April 2022,^[7]^ with sample sizes ranging from 36^[24]^ to 841.^[7]^ Ten trials^[7,11,16–19,22–25]^ used melatonin for delirium prevention and 2 trials^[20,21]^ used ramelteon. The dosage and course of each trial are presented in Table 1. No placebo was used as a control in 1 trial by Mahrose,^[25]^ and placebo was used in the remaining trials. A trial conducted by Nickkholgh et al^[24]^ used a single high dose of melatonin (50 mg/kg). Six trials exclusively included surgical patients admitted to the ICU. Of these trials, 1 trial^[16]^ included elderly patients with acute heart failure after surgery and did not explicitly report the type of surgery. In the remaining 5 trials, patients underwent liver resection,^[24]^ elective on-pump coronary artery bypass graft surgery,^[19,25]^ elective pulmonary thromboendarterectomy surgery,^[20]^ and percutaneous transluminal coronary intervention surgery.^[18]^ The main patient characteristics of the 6 trials, including surgical patients, are summarized in Table 2. Two trials^[21,23]^ included patients admitted to the medical or emergency ICU. The trial^[23]^ conducted by Vijayakumar et al exclusively included patients with organophosphorus compound poisoning. Four trials^[7,11,17,22]^ included mixed medical and surgical ICU patients. The main patient characteristics of the 6 trials, including medical and mixed patients, are summarized in Table 3.

**Table 1:** Characteristics of included trials.

Study	Country	Population	Sample size (Melatonin /Placebo)	Type of melatonin	Placebo	Melatonin administration	ICU Delirium Assessment Tool	Risk of Bias
Nickkholgh 2011	Germany	Surgical patients Liver resection	36(18/18)	Melatonin	Yes	Single dose of melatonin (50 mg/kg) dissolved in 250 mL of milk after the intubation for general anesthesia	NR	Some concern
Vijayakumar 2016	India	Medical patient OPCP	56(26/30)	Melatonin	Yes	3 mg of melatonin at 9 pm until discharged from ICU	CAM-ICU	Low
Nishikimi 2018	Japan	EMICU patients	88(45/43)	Ramelteon	Yes	8 mg of ramelteon at 8 pm until discharged from ICU	CAM-ICU	Low
Abbasi 2018	Iran	Mixed patients	137(67/70)	Melatonin	Yes	3 mg of melatonin at 9 pm for 5 d	CAM-ICU	Some concern
Jaiswal 2019	USA	Surgical patients PTE	117(59/58)	Ramelteon	Yes	8 mg of ramelteon at 8 pm for 6 d (beginning the night prior the surgery for a maximum of 7 nights) or until ICU discharge	CAM-ICU	Low
Gandolfi 2020	Brazil	Mixed patients	203(102/101)	Melatonin	Yes	10 mg of melatonin at 8 pm for 7 d	ICDSC	Low
Zadeh 2021	Iran	Surgical patient CABG	60(30/30)	Melatonin	Yes	3 mg of melatonin for 3 d (the evening before the operation; the morning of surgery, and second postoperative day)	CAM-ICU	Low
Shi 2021	China	Surgical patient PCI	297(148/149)	Melatonin	Yes	3 mg/d melatonin for 7 d after PCI	CAM-ICU	High
Mahrose 2021	Egypt	Surgical patient CABG	110(55/55)	Melatonin	No	5 mg of melatonin in the night before surgery and same dose was repeated every 24 hours for 3 postoperative days	CAM-ICU	High
Yin 2022	China	Surgical patient AIH	497(248/249)	Melatonin	Yes	3 mg/d melatonin for 7 d after acute AIH	CAM-ICU	High
Behdani 2022	Iran	Mixed patient SIH	96(48/48)	Melatonin	Yes	Melatonin tablet 3 mg twice daily (12 am and 12 pm) each time 2 tablets for 3 d	RASS	High
Wibrow 2022	Australian	Mixed patients	841(419/422)	Melatonin	Yes	4 mg of melatonin at 9 pm for 14 consecutive nights or until ICU discharge	CAM-ICU	Low

AIH = acute heart failure, CABG = coronary artery bypass graft, CAM-ICU = Confusion Assessment Method for ICU, EMICU = emergency and medical ICU, ICDSC = Intensive Care Delirium Screening Checklist, ICU = intensive care unit, NR = not report, OPCP = organophosphorus compound poisoning, PCI = percutaneous transluminal coronary intervention, PTE = pulmonary thromboendarterectomy, RASS = Richmond Agitation Sedation Scale, SIH = stress induced hyperglycemia.

**Table 2:** Patient characteristics of included studies enrolling surgical ICU patients.

Patient characteristics	Nickkholgh 2011 (N = 36)	Jaiswal 2019 (N = 117)	Zadeh 2021 (N = 60)	Shi 2021 (N = 297)	Mahrose 2021 (N = 110)	Yin 2022 (N = 497)
Age—yrs, mean	57.5	57.1	61.6	71.6	66.6	68.8
Sex—male, n (%)	28(77.8)	58(49.6)	42(70)	182(61.3)	82(74.5)	295(59.4)
BMI, mean	25.1	32.1	NR	NR	NR	NR
Smoker, n (%)	NR	NR	22(36.7)	94(31.6)	54(49.1)	140(28.2)
Regular alcohol use, n (%)	NR	NR	0	NR		NR
Type of surgery, n (%)	Liver resection	PTE	Elective on-pump CABG	PCI	CABG	NR
Type of anesthesia	General anesthesia	General anesthesia	General anesthesia	General anesthesia	General anesthesia	NR
Duration of operation [min]	207	517.9	NR	132	NR	NR

BMI = body mass index, CABG = coronary artery bypass graft, ICU = intensive care unit, NR = not report, PCI = percutaneous transluminal coronary intervention, PTE = pulmonary thromboendarterectomy.

**Table 3:** Patient characteristics of included studies enrolling medical ICU and mixed ICU patients.

Patient characteristics	Vijayakumar2016 (N = 56)	Nishikimi 2018 (N = 88)	Abbasi 2018 (N = 137)	Gandolfi 2020 (N = 203)	Wibrow 2022 (N = 841)	Behdani2022 (N = 96)
Age—yrs, mean	37.3	68	51.2	61	61.9	56
Sex—male, n (%)	37(66.1)	57(64.8)	78(56.9)	117(57.6)	527 (62.7)	62(64.6)
BMI, mean	23.4	NR	NR	NR	NR	25.7
Smoker, n (%)	NR	NR	NR	NR	179(21.3）	NR
Regular alcohol use, n (%)	NR	4(4.5)	NR	NR	305(36.3)	NR
APACHE-II sore, mean	9.32	23.9	7.7	NR	17.4	12
SOFA sore, mean	NR	8.3	3.2	NR	6.1	NR
Transferred from, n (%)						
Surgical	NR	NR	80(58.4)	91(44.8)	213(25.3)	NR
Medical	NR	NR	34(24.8)	23(11.2)	388(46.1)	NR
Other	NR	NR	23(16.8)	89(44)	240(28.6)	NR
Diagnosis type, n (%)						
Cardiovascular	–	20(22.7)	43(31.4)	61(30.1)	193(22.9)	10(10.4)
Respiratory	–	18(20.5)	2(1.5)	7(3.4)	228(27.1)	–
Neurological	–	–	10(7.3)	13(6.4)	46(5.5)	–
Sepsis	–	21(23.9)	–	–	198(23.5)	–
Trauma	–	–	–	–	66(7.9)	35(36.5)
TBI	–	–	–	–	2(0.2)	18(18.8)
Other	OPCP: 56(100)	29(32.9)		13(6.5)	108(12.9)	33(34.3)

APACHE-II = Acute Physiology and Chronic Health Evaluation II, BMI = body mass index, ICU = intensive care unit, NR = not report, OPCP = organophosphate compound poisoning, SOFA = Sequential Organ Failure Assessment, TBI = Traumatic brain injury.

### 3.2. Risk of bias

The risk of bias assessment for all included studies is shown in Figure S1, Supplemental Digital Content, http://links.lww.com/MD/H804. Four RCTs^[16,17,22,25]^ were deemed to have a high risk of bias in the overall assessment, 2 RCTs^[22,24]^ were deemed to have some concern of bias, and 6 RCTs^[7,11,19–21,23]^ were rated as having a low risk of bias overall. A funnel plot of the primary outcome showed no publication bias (see Figure S2, Supplemental Digital Content, http://links.lww.com/MD/H805).

### 3.3. The primary outcome

The results of the primary and secondary outcomes are displayed in Table 4. The reported prevalence of ICU delirium varied from 2.78%^[24]^ to 67.86%.^[23]^ We found that melatonin reduced the prevalence of ICU delirium (RR, 0.77; 95% CI: 0.61–0.96; *I*^2^ = 56%). When all the trials were pooled, significant heterogeneity was observed. We excluded studies individually, but there was still significant heterogeneity. In the subgroup analyses, melatonin was associated with a significant reduction in delirium occurrence in surgical (RR, 0.69; 95% CI: 0.56–0.86; *I*^2^ = 13%) and medical patients (RR, 0.57; 95% CI: 0.40–0.80; *I*^2^ = 0%). In our older patients aged ≥ 60 years, 5 trials with 956 patients provided data. Of these, 3 trials^[16,18,25]^ included only older patients aged > 60 years, 1 trial^[19]^ conducted by Jaiswal et al reported the incidence of delirium in patients aged ≥ 65 years, and the remaining 1^[19]^ by Zadeh et al reported the incidence of delirium in patients aged > 70 years. Melatonin reduced the occurrence of delirium in older patients (RR, 0.69; 95% CI: 0.57–0.83; *I*^2^ = 0%). In the cardiovascular disease subgroup, 5 trials,^[16,18–20,25]^ including 1081 patients, provided data. Melatonin did reduce the delirium occurrence in cardiovascular disease patients with diagnoses of cardiovascular diseases (RR, 0.70; 95% CI: 0.58–0.84; *I*^2^ = 0%). There was no significant difference in delirium occurrence between the groups in the melatonin subgroup (RR, 0.79; 95% CI: 0.61–1.02; *I*^2^ = 60%) and in the ramelteon subgroup (RR, 0.69; 95% CI: 0.43–1.10; *I*^2 ^= 31%). However, the result of the sensitivity analysis, with 6 RCTs^[7,11,19–21,23]^ at low risk of bias, was inconsistent with the primary analysis (RR, 0.74; 95% CI: 0.52–1.05; *I*^2^ = 63%).

**Table 4:** Data analysis of primary and secondary outcomes.

	Outcomes	Trials	Simple size	RR/MD	95% CI	*I*^2^(%)	*P*
1	Delirium prevalence	12	2538	0.77	0.61 to 0.96	56	.02
1.1	Subgroup analyses by ICU population						
	Surgical patients	7	1197	0.69	0.56 to 0.86	13	.0009
	Medical patients	3	178	0.57	0.40 to 0.80	0	.001
	Mixed patients	4	1163	1.11	0.93 to 1.32	0	.24
1.2	Subgroup analysis of older patients	5	956	0.69	0.57 to 0.83	0	<.0001
1.3	Subgroup analysis of cardiovascular disease patients	5	1081	0.70	0.58 to 0.84	0	<.0001
1.4	Subgroup analyses by melatonin types						
	Melatonin	10	2333	0.79	0.61 to 1.02	60	.07
	Ramelteon	2	205	0.69	0.43 to 1.10	31	.12
1.5	Sensitivity analysis (studies at low risk of bias)	6	1365	0.74	0.52 to 1.05	63	.09
2	Duration of delirium	4	452	−0.11	−1.01 to 0.78	96	.80
3	Duration of MV	6	1387	-0.02	−0.07 to 0.02	33	.32
4	ICU LOS	10	1744	−0.16	−0.70 to 0.38	60	.57
5	Hospital LOS	8	2238	−0.75	−2.27 to 0.77	85	.33
6	Mortality	10	2350	0.90	0.77 to 1.06	0	.20
7	Sleep quality						
	Very poor	2	1033	0.54	0.11 to 2.62	85	.44
	Poor	2	1033	1.06	0.84 to 1.36	0	.61
	Good	2	1033	0.94	0.74 to 1.19	0	.60
	Very good	2	1033	1.31	0.97 to 1.78	0	.08

CI = confidence interval; ICU intensive care unit, LOS = length of stay, MD = mean difference, MV = mechanical ventilation, RR = relative risk.

**Figure 2.**
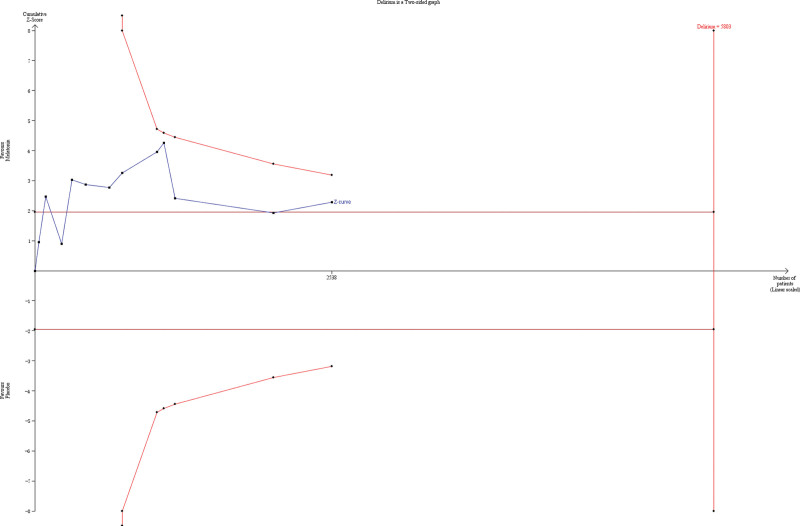
TSA of incidence of delirium. A diversity adjusted information size of 5803 patients was calculated using 5% of type 1 error (2-sided), a power of 80%, an anticipated relative risk of 20.0%, and delirium incidence of 31.60% in the control arm. TSA = trial sequential analysis.

**Figure 3.**
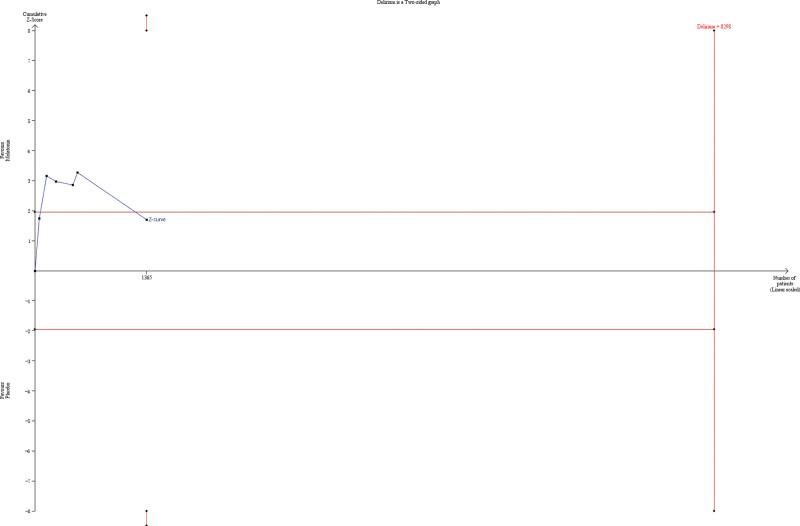
TSA of incidence of delirium including RCTs at low risk of bias. A diversity adjusted information size of 8298 patients was calculated using 5% of type 1 error (2-sided), a power of 80%, an anticipated relative risk of 20.0%, and delirium incidence of 31.60% in the control arm. TSA = trial sequential analysis.

TSA was performed to determine delirium prevalence, the primary outcome. TSA indicated that the Z-curve crossed the traditional boundary; however, it did not cross the monitoring boundary for benefit, indicating that it is still inconclusive that melatonin affects delirium incidence. Only 43.74% of the required sample size (5803 patients) was accrued in the current analysis (Fig. 2). In addition, TSA, including RCTs with a low risk of bias, was performed on the primary outcome. A diversity-adjusted information size of 8298 patients was calculated using 5% of type 1 error (2-sided), a power of 80%, an anticipated relative risk of 20.0%, and delirium incidence of 31.60% in the control arm. Only 16.45% of the required sample size was accrued in the current analysis, which was consistent with the result of TSA, including all trials (Fig. 3).

### 3.4. Second outcomes

#### 3.4.1. Duration of delirium and delirium-free day.

Four trials^[20–22,25]^ reported data on delirium duration. No significant difference was observed in delirium duration (MD, −0.11; 95% CI: −1.01 to 0.78; *I*^2^ = 96%).

In addition, Vijayakumar et al^[23]^ reported that delirium-free time was significantly shorter in the melatonin group than in the placebo group (*P* = .001). Jaiswal et al^[20]^ showed that no differences in delirium/coma-free days (*P* = .181) or coma-free days (*P* = .210) between the groups. Wibrow et al^[7]^ reported no significant difference in the average proportion of delirium-free assessments per patient between the melatonin and placebo groups (*P* = .547) and no difference in the total number of patients free from delirium or coma throughout the study (*P* = .137).

#### 3.4.2. Serum melatonin concentration.

Nickkholgh et al^[24]^ reported that melatonin was effectively absorbed with serum concentrations of 1142.8 ± 7.2 ng/mL (mean ± standard error of mean) versus 0.3 ± 7.8 ng/mL in controls (*P* < .0001). Gandolfi et al^[11]^ reported that the median serum melatonin concentration was 150 pg/mL (range, 125–2125) in the melatonin group compared with 32.5 pg/mL (18.5–35) in the placebo group (*P* < .001) at 2:00 am (7 hours after administration). Wibrow et al^[7]^ reported that the median serum melatonin concentration 2 to 3 hours after administration was 19.4 ng/mL in the melatonin group and < 0.1 ng/mL in the placebo group.

#### 3.4.3. Need for sedation.

Vijayakumar et al^[23]^ reported that the requirement for midazolam (*P* < .001) and fentanyl (*P* = .03) decreased significantly in the melatonin group. Jaiswal et al^[20]^ showed that a similar number of delirious and non-delirious participants received postoperative benzodiazepines, and the doses were not different between the groups. Gandolfi et al^[11]^ reported no significant statistical difference regarding the days free and mean daily doses of analgesics and sedatives between groups. Wibrow et al^[7]^ reported no differences in the use of benzodiazepines or physical restraints.

#### 3.4.4. Duration of MV.

Six trials^[7,11,19,20,23,25]^ with 1387 patients reported data on the duration of MV. There was no difference in the duration of MV between the melatonin and placebo groups (MD, −0.02; 95% CI, −0.07 − 0.02; *I*^2^ = 33%). In addition, Behdani et al^[17]^ showed that melatonin did not improve ventilator dependence (*P* > .2).

#### 3.4.5. ICU and hospital LOS.

Ten trials^[7,11,17,19–25]^ including 1744 patients reported data on ICU LOS, and 8 trials^[7,11,16,18,20,22,24,25]^ including 2238 patients reported data on hospital LOS. The ICU LOS was 0.16 days shorter among patients who received melatonin compared with those in the placebo group, but the outcome was not statistically significant (MD, −0.16; 95% CI: −0.70 to 0.38; *I*^2 ^= 60%). Regarding hospital LOS, no significant difference was found between the groups (MD, −0.75; 95% CI: −2.27 to 0.77; *I*^2 ^= 85%).

### 3.5. Mortality

Ten studies^[7,11,16–18,20–24]^ with a total of 2350 patients reported mortality as an outcome. We found no significant difference in mortality between patients receiving melatonin and those receiving placebo (RR, 0.90; 95% CI: 0.77–1.06; *I*^2 ^= 0%).

### 3.6. Sleep quality

In our meta-analysis, only 2 trials^[7,11]^ reported data on sleep quality. Nocturnal sleep quality was evaluated using the Richards Campbell sleep questionnaire.^[26]^ The scores ranged from 0, indicating the worst possible sleep, to 100, indicating the best possible sleep. The total Richards Campbell sleep questionnaire sleep score was derived by summing the individual scores of the 5 sleep items and dividing it by 5. Participants with scores of 1 to 25 were considered to have very poor sleep, scores 26 to 50, poor sleep, scores 51 to 75, good sleep, and scores 76 to 100, very good sleep. Melatonin did not improve sleep quality compared to placebo.

### 3.7. Adverse events

Four trials^[7,19,23,24]^ reported no treatment-related adverse events. One trial conducted by Behdani et al^[17]^ reported that the only side effect that could be attributed to melatonin was pruritus, and this event was only seen in 1 patient in the melatonin group. One trial^[11]^ reported that 63 patients had adverse events considered as any untoward medical occurrence in a patient enrolled in this study, regardless of its causal relationship to melatonin.

## 4. Discussion

In this meta-analysis, we included 12 trials with 2538 adult ICU patients to evaluate the effectiveness of prophylactic melatonin in delirium prevention. We found that prophylactic melatonin may reduce the prevalence of delirium compared with placebo. However, these findings should be interpreted with caution. The results of sensitivity analysis and TSA did not support the primary outcomes.

Our meta-analysis findings were inconsistent with the latest RCT^[7]^ by Wibrow et al, which included mixed patients. In addition, most RCTs^[7,11,20]^ with a low risk of bias, including mixed patients, did not support that melatonin reduces delirium prevalence.^[27–29]^ First, ICU patients are heterogeneous and suffer from different diseases with multiple concurrent problems. Due to the various clinical populations, illness severity, and heterogeneous diagnoses, it is difficult to demonstrate the benefits of melatonin on delirium. In our meta-analysis, the trials, including only surgical or medical patients, showed that melatonin might decrease delirium prevalence. A trial by Shi et al,^[18]^ enrolling older patients undergoing percutaneous transluminal coronary intervention surgery, demonstrated that melatonin significantly reduced the delirium prevalence. One trial by Vijayakumar et al^[23]^ included adult patients with organophosphorus compound poisoning and demonstrated that melatonin reduced delirium prevalence. In our subgroup analyses, patients in the surgical and medical ICU may benefit from melatonin for delirium prevention, but patients in mixed ICU. In addition, we found that older patients and those with cardiovascular disease may benefit from melatonin in our subgroup. Therefore, it is essential to evaluate which specific ICU patients would most likely benefit from melatonin.

Second, any potential benefits of melatonin may be more evident in this population with a high risk of delirium.^[7]^ Multiple risk factors collectively contribute to the onset and persistence of ICU delirium, including pain, deep sedation, use of MV, analgesics and sedatives, and immobility.^[30–32]^ Although critically ill patients are at a high risk of delirium, not every ICU patient has all the above-mentioned risk factors or a similar degree of risk. Delirium occurs in 60–80% of mechanically ventilated ICU patients and 20% to 50% of nonventilated patients.^[30]^ Various clinical populations with different risk factors may make it difficult to demonstrate the benefits of melatonin on delirium. Moreover, there are several differences in delirium prevention and treatment between different ICUs. Clinical guidelines, including the pain, agitation, delirium, immobility, and sleep guidelines, have recommended using a bundle approach, such as the “ABCDEF bundle,” to target eliminating multiple modifiable risk factors of ICU delirium to reduce the chances of or shorten delirium duration in critically ill adults.^[32–34]^ Clinical guidelines have considered bundle interventions that include non-pharmacological interventions to decrease the prevalence and shorten the duration of ICU delirium. Therefore, it is difficult to determine whether melatonin has an effect on ICU patients. It is difficult for every center to obtain information about other delirium-prevention intervention strategies, especially non-pharmacological strategies. Early melatonin administration may be practiced based on a bundle approach for ICU delirium prevention. Further studies are needed to examine the effectiveness of prophylactic melatonin based on the bundle approach on the prevalence and duration of ICU delirium.

However, the results of this meta-analysis should be interpreted with caution. The results of TSA neither support nor oppose the use of melatonin to prevent ICU delirium. In other words, the present results may have false-positive errors, and future large-scale rigorous randomized trials with better designs are needed to provide more certainty regarding the use of melatonin to prevent ICU delirium.

Our meta-analysis has several limitations. First, in our meta-analysis, significant heterogeneity was found in the pooled results for delirium occurrence, delirium duration, ICU LOS, and hospital LOS. A possible reason for this heterogeneity could be the different ICU populations. Second, the number of studies in the current analysis reporting outcomes on delirium duration and delirium-free status was small. Only 3 trials reported data on delirium duration. There was significant heterogeneity in the pooled results. We also reported, in narrative form, relevant delirium-free data. Delirium duration and a delirium-free status are important markers of delirium prevalence. Reporting delirium prevalence alone was not sufficient to evaluate the efficacy of melatonin in delirium prevention. Only 2 trials reported data on sleep quality that was not large enough to allow analysis of the association between melatonin and sleep. Third, our meta-analysis failed to evaluate the efficacy of melatonin based on different disease diagnoses, such as sepsis and respiratory diseases. Although we conducted a cardiovascular disease subgroup analysis, the majority of the patients with cardiovascular disease were from surgical ICU. Trials from mixed ICU also enrolled cardiovascular disease patients, but we did not obtain specific data on cardiovascular disease outcomes from these trials. Therefore, the results of our cardiovascular disease subgroup may have limited generalizability. Fourth, analgesics, co-sedatives, agitation, and non-pharmacological treatments may have had confounding effects. Fifth, some trials did include ICU patients but not in detail and we potentially excluded them, so the introduction of selection bias is a possibility. Finally, we could not exclude the possibility of publication bias because we did not include unpublished trials or full-text articles.

## 5. Conclusions

This meta-analysis found that the early administration of melatonin may decrease delirium prevalence in critically ill patients. However, sensitivity analysis of high-quality studies did not support this finding. In addition, TSA demonstrated that the results might have false-positive errors. Therefore, these findings should be cautiously interpreted. Further studies are needed to examine the effectiveness of prophylactic melatonin on the prevalence and duration of ICU delirium.

## Author contributions

**Conceptualization:** Chen Li, Zhi Chen.

**Data curation:** Wenqiang Zhou.

**Funding acquisition:** Zhi Chen.

**Methodology:** Wenqing Yan, Chen Li, Xin Song, Wenqiang Zhou, Zhi Chen.

**Software:** Wenqing Yan,

**Visualization:** Zhi Chen.

**Writing – original draft:** Wenqing Yan, Chen Li.

**Writing – review & editing:** Wenqing Yan, Xin Song, Zhi Chen.

## Supplementary Material


