# Supplementary material for: Prophylactic melatonin for delirium in critically ill patients: A systematic review and meta-analysis with trial sequential analysis
Source: Medicine (Baltimore). 2022 Oct 28;101(43):e31411. doi: 10.1097/MD.0000000000031411 (PMC9622662; doi:10.1097/MD.0000000000031411)
Supplement: Supplementary file 1 [file medi-101-e31411-s001.pdf]

**Prophylactic melatonin for delirium in critically ill patients: a systematic review and meta-analysis with trial sequential analysis**

Wenqing Yan; Chen Li; Xin Song; Wenqiang Zhou; Zhi Chen，M.D.

Supplement 1 Search strategy

PubMed (1796 to April 15, 2022)

| Step | Search String |
|---|---|
| 1 | "Delirium"[Mesh] OR delirium OR confusion OR acute confusional syndrome OR postoperative delirium OR cognitive dysfunction OR ICU delirium OR ICU psychosis OR ICU syndrome OR deliri* |
| 2 | "Melatonin"[Mesh] OR Melatonin OR melatonergic OR ramelteon OR melatonin receptor agonist OR melatonin MT1 receptors agonist OR melatonin MT2 receptors agonist |
| 3 | "Intensive Care Units"[Mesh] OR critically ill OR critical illness OR critical care OR intensive care OR critically ill patient OR intensive care unit OR ICU |
| 4 | 1 AND 2 AND 3 |

Embase (1974 to April 15, 2022)

| Step | Search String |
|---|---|
| 1 | 'delirium'/exp OR delirium OR 'confusion'/exp OR confusion OR 'acute confusional syndrome' OR (acute AND confusional AND ('syndrome'/exp OR syndrome)) OR 'postoperative delirium'/exp OR 'postoperative delirium' OR (postoperative AND ('delirium'/exp OR delirium)) OR 'cognitive dysfunction'/exp OR 'cognitive dysfunction' OR (cognitive AND dysfunction) OR 'icu delirium'/exp OR 'icu delirium' OR (icu AND ('delirium'/exp OR delirium)) OR 'icu psychosis'/exp OR 'icu psychosis' OR (icu AND ('psychosis'/exp OR psychosis)) OR 'icu syndrome' OR (icu AND ('syndrome'/exp OR syndrome)) |
| 2 | 'melatonin'/exp OR melatonin OR melatonergic OR 'ramelteon'/exp OR ramelteon OR 'melatonin receptor agonist'/exp OR 'melatonin receptor agonist' OR (('melatonin'/exp OR melatonin) AND ('receptor'/exp OR receptor) AND ('agonist'/exp OR agonist)) OR 'melatonin mt1 receptors agonist' OR (('melatonin'/exp OR melatonin) AND mt1 AND ('receptors'/exp OR receptors) AND ('agonist'/exp OR agonist)) OR 'melatonin mt2 receptors agonist' OR (('melatonin'/exp OR melatonin) AND mt2 AND ('receptors'/exp OR receptors) AND ('agonist'/exp OR agonist)) |
| 3 | 'critically ill'/exp OR 'critically ill' OR (critically AND ill) OR 'critical illness'/exp OR 'critical illness' OR (critical AND ('illness'/exp OR illness)) OR 'critical care'/exp OR 'critical care' OR (critical AND ('care'/exp OR care)) OR 'intensive care'/exp OR 'intensive care' OR (intensive AND ('care'/exp OR care)) OR 'critically ill patient'/exp OR 'critically ill patient' OR (critically AND ill AND ('patient'/exp OR patient)) OR 'intensive care unit'/exp OR 'intensive care unit' OR (intensive AND ('care'/exp OR care) AND ('unit'/exp OR unit)) OR icu |
| 4 | 1 AND 2 AND 3 |
