# Supplementary material for: Prophylactic melatonin for delirium in critically ill patients: A systematic review and meta-analysis with trial sequential analysis
Source: Medicine (Baltimore). 2022 Oct 28;101(43):e31411. doi: 10.1097/MD.0000000000031411 (PMC9622662; doi:10.1097/MD.0000000000031411)
Supplement: Supplementary file 2 [file medi-101-e31411-s002.pdf]

Prophylactic melatonin for delirium in critically ill patients: a systematic review and meta-analysis with trial sequential analysis

Wenqing Yan; Chen Li; Xin Song; Wenqiang Zhou; Zhi Chen，M.D.

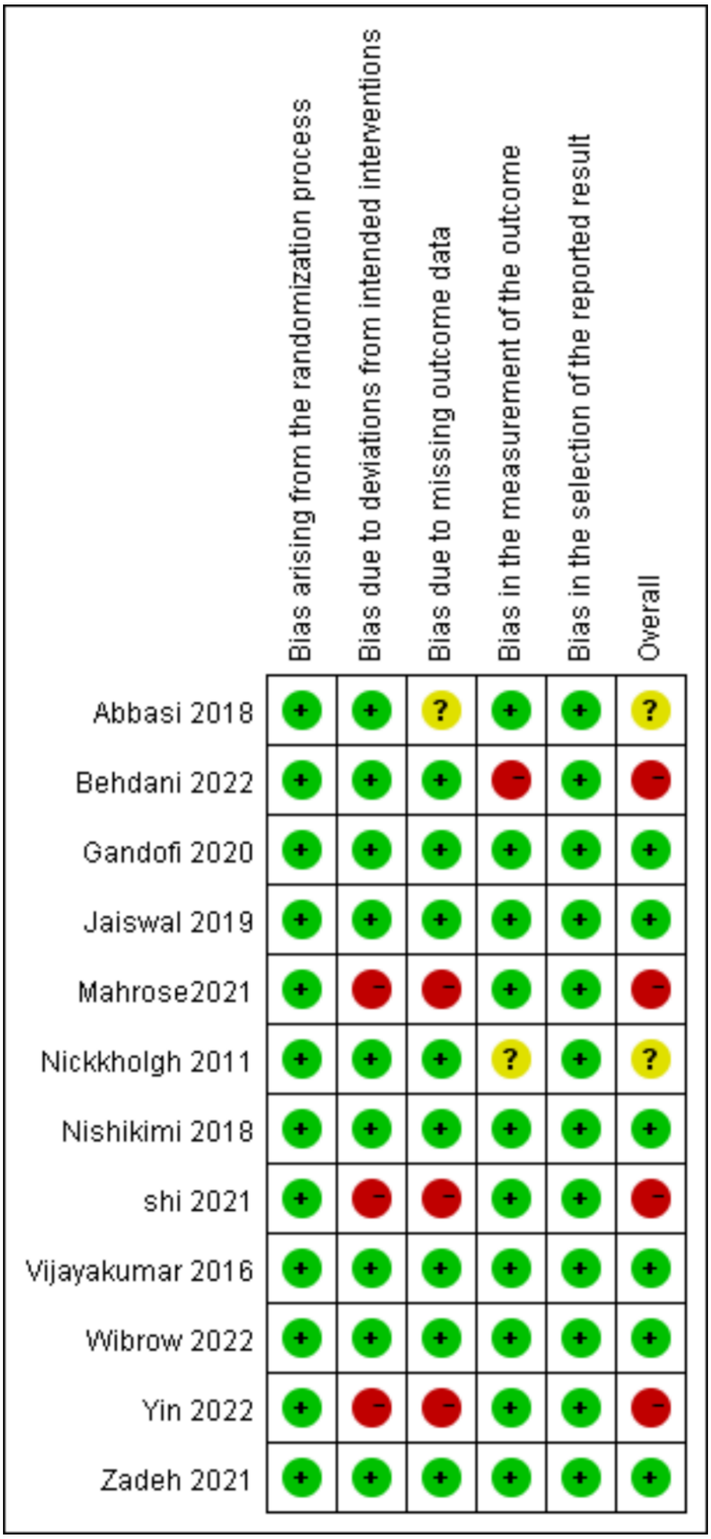

Supplement Figure 1 Risk of bias summary.
